# ‘Antibiotic footprint’ as a communication tool to aid reduction of antibiotic consumption

**DOI:** 10.1093/jac/dkz185

**Published:** 2019-05-10

**Authors:** Direk Limmathurotsakul, Jonathan A T Sandoe, David C Barrett, Michael Corley, Li Yang Hsu, Marc Mendelson, Peter Collignon, Ramanan Laxminarayan, Sharon J Peacock, Philip Howard

**Affiliations:** 1 Mahidol-Oxford Tropical Medicine Research Unit, Faculty of Tropical Medicine, Mahidol University, Bangkok, 10400, Thailand; 2 Department of Tropical Hygiene, Faculty of Tropical Medicine, Mahidol University, Bangkok, 10400, Thailand; 3 Centre for Tropical Medicine and Global Health, University of Oxford, Oxford OX3 7FZ, UK; 4 University of Leeds/Leeds Teaching Hospitals NHS Trust, Leeds LS1 3EX, UK; 5 British Society of Antimicrobial Chemotherapy, Birmingham B1 3NJ, UK; 6 Bristol Veterinary School, University of Bristol, Bristol BS40 5DU, UK; 7 Saw Swee Hock School of Public Health, National University of Singapore and National University Health System, 12 Science Drive 2, Singapore 117649, Singapore; 8 National Centre for Infectious Diseases, Moulmein Road, Singapore 308433, Singapore; 9 Division of Infectious Diseases & HIV Medicine, Department of Medicine, University of Cape Town, Cape Town, 7925, South Africa; 10 International Society for Infectious Diseases, Brookline, MA 02446, USA; 11 Infectious Diseases and Microbiology, Canberra Hospital, Canberra, 2605, Australia; 12 Medical School, Australian National University, Acton, 2606, Australia; 13 Center for Disease Dynamics, Economics & Policy, New Delhi, 110024, India; 14 Princeton Environmental Institute, Princeton, NJ 08544, USA; 15 Department of Medicine, University of Cambridge, Cambridge CB2 0QQ, UK

## Abstract

‘Superbugs’, bacteria that have become resistant to antibiotics, have been in numerous media headlines, raising awareness of antibiotic resistance and leading to multiple action plans from policymakers worldwide. However, many commonly used terms, such as ‘the war against superbugs’, risk misleading people to request ‘new’ or ‘stronger’ antibiotics from their doctors, veterinary surgeons or pharmacists, rather than addressing a fundamental issue: the misuse and overuse of antibiotics in humans and animals. Simple measures of antibiotic consumption are needed for mass communication. In this article, we describe the concept of the ‘antibiotic footprint’ as a tool to communicate to the public the magnitude of antibiotic use in humans, animals and industry, and how it could support the reduction of overuse and misuse of antibiotics worldwide. We propose that people need to make appropriate changes in behaviour that reduce their direct and indirect consumption of antibiotics.

## Introduction

Antibiotic resistance is an increasingly serious threat to public health. When bacteria can stop an antibiotic from working against them, standard antibiotic treatments become ineffective and infections with antibiotic-resistant bacteria are associated with a higher risk of death. Hundreds of thousands of deaths per year are estimated to be attributable to antibiotic resistance.^1^ That number is likely to rise to many millions per year by 2050. The misuse and overuse of antibiotics is a key contributor to this problem. Antibiotics consumed by humans and animals are often excreted as active drugs (that is, the body does not deactivate them), and these enter the sewage systems and water sources, where they select for antibiotic-resistant bacteria in the environment.^1^

International health organizations encourage all countries to reduce their use of antibiotics in both humans and animals to a minimum, but limited public understanding of antibiotic resistance and its drivers is likely to be a major barrier to the reduction of inappropriate antibiotic use.^2^ Antibiotics are often inappropriately used to treat viral infections in humans, including the common cold. A large number of people worldwide incorrectly believe that antibiotics are effective for the common cold and influenza-like illnesses.^3^^,^^4^ A recent study in the UK found that antibiotic resistance information given to participants with low awareness could, paradoxically, lead them to ask a doctor for antibiotics more often.^5^ Therefore, communication messages on antibiotic resistance should be simple,^5^ and individuals need to be supported to understand that appropriate behaviour change is important to them personally.^6^ In addition, antibiotics are used in large quantities in agriculture to maintain animal health, and in industry and household products for reasons largely unrelated to human health.^7^ Consumers are in a strong position to influence the use of antibiotics everywhere. But access to usage volumes and usage patterns is limited and consumers are poorly informed about existing use, which reduces the impetus for change.

## Antibiotic footprint

The ‘antibiotic footprint’ has been proposed as a global tool for the public communication of the magnitude of antibiotic use in humans, animals and industry,^8^ which could build on the success of the concept of the carbon footprint. People need to use energy to live, but using too much energy has been driving climate change globally. Likewise, people and animals need antibiotics if they are infected with bacteria. However, overuse and misuse of antibiotics in humans and animals are fostering antibiotic-resistant bacteria, and will increase the global number of human and animal deaths they cause over time.^1^ The antibiotic footprint could serve as a tool to better inform people of the widespread and often unnecessary use of antibiotics, which could help to reduce their use locally and globally, and in turn reduce the risk of antibiotic-resistant bacteria and, potentially, infections with antibiotic-resistant bacteria. Below, we discuss the similarity between the antibiotic footprint and the carbon footprint, and potential uses of the antibiotic footprint.

## Carbon footprint as a model

Just as reducing the carbon footprint to a minimum is the goal, the aim of the antibiotic footprint would be to reduce antibiotic consumption to a minimum. Clear ways of communicating this to the public are important in tackling both climate change and antibiotic resistance. Formerly, the research community used life cycle assessment (LCA) as an indicator of climate change potential.^9^ LCA was intended to create a holistic picture, provide information about the consequences of change, and inform policy makers. Subsequently, the term ‘carbon footprint’ was adopted, mainly by pro-active non-governmental organizations (NGOs) and the private sector. This concept is catchy, simple to understand and easy to calculate on-line, and retail chains and companies proactively request and provide such information to consumers.^9^

**Figure 1.**
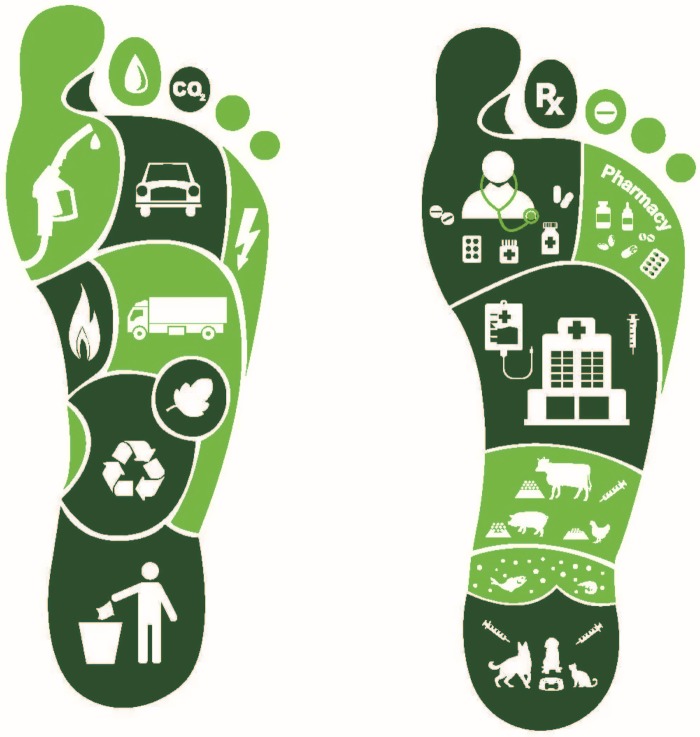
A conceptual figure for carbon footprint and antibiotic footprint. The figure shows a holistic approach to the carbon footprint (left) and antibiotic footprint (right). The antibiotic footprint is defined as the total amount of antibiotic consumption that is associated with human activities, including direct consumption of antibiotics by humans at community and hospital levels and consumption by animals. The figure is reproduced from www.antibioticfootprint.net under the terms of the Creative Commons Attribution 4.0. This figure appears in colour in the online version of *JAC* and in black and white in the print version of *JAC*.

As a carbon footprint measures the quantity of gaseous emissions relevant to climate change and is associated with activities such as automobile use, building heating and agriculture (Figure 1), an antibiotic footprint can be used to attribute antibiotic consumption to different human activities. This includes direct consumption of antibiotics at community and hospital levels, and indirect consumption, for example via animals bred for food. The antibiotic footprint also aligns with the concept of One Health since the antibiotic footprint considers antibiotic consumption in all sectors, including humans and animals.

Simple measures of consumption are needed; carbon emissions are measured in units of mass of carbon dioxide and antibiotic consumption could be measured in units of mass of antibiotics as a total value or per head of population. Currently, many indicators are proposed and used for antibiotic consumption in human and animals, including the DDD per 1000 inhabitants per day and milligrams per population correction unit (PCU).^10^^,^^11^ These terms were created to support fair comparisons among different antibiotics and different animals included in the calculation, but are complex terms. Learning from the carbon footprint, it is likely to be better to design the public communication strategy around antibiotic consumption in simple terms of mass of antibiotics.

Nation-level carbon footprint data are now widely and openly available, with tools for their easy visualization. In comparison, the majority of national data across all sectors (humans and animals) on antibiotic consumption come from high-income countries.^12–14^ National data on antibiotic consumption for human use in low- and middle-income countries (LMICs) are becoming increasingly available,^15^ but national data on consumption for animal use in LMICs is rarely available.^16^ Complete data on antibiotic usage in each sector should be made openly available so that multiple metrics (such as total consumption, DDD, mg/PCU, mg/kg, daily dose metrics and course dose metrics)^10^^,^^11^ of each antibiotic (particularly of the highest-priority critically important antibiotics)^17^ can be estimated and used by clinicians, farmers, researchers and policymakers. Then, the increasing information on complete antibiotic use in each country (and all metrics and research evidence on antibiotic resistance) would allow the communication strategy to be adjusted and improved over time.

## Potential uses of antibiotic footprint

**Figure 2.**
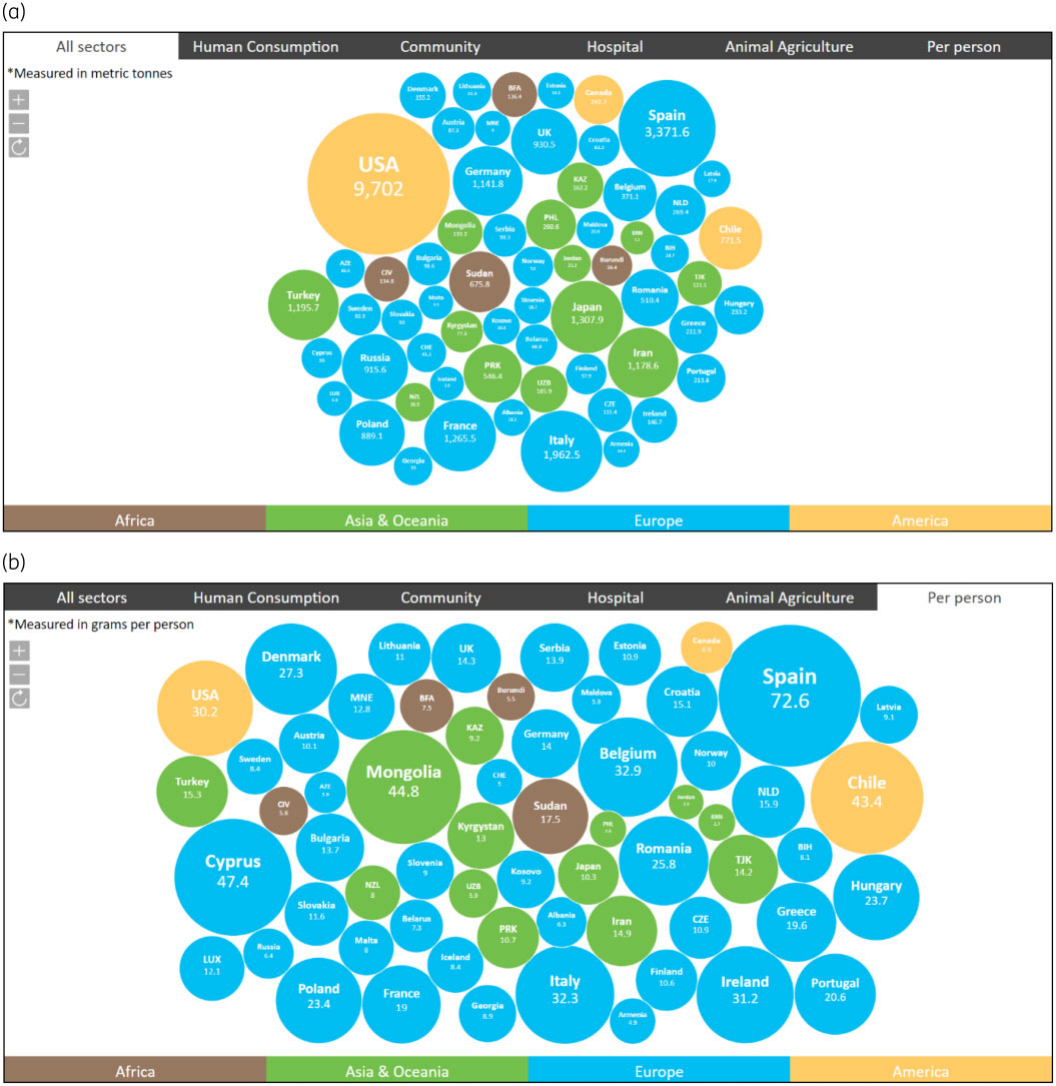
Examples of antibiotic footprint (a) by country (metric tonnes) and (b) per capita (grams per person) in 2015 based on official and open-access data for the countries shown. Only official and open-access data from each country were used. Antibiotic consumption data in humans were based on open-access official data in the WHO Report on Surveillance of Antibiotic Consumption.^15^ Data on antibiotic consumption in animal agriculture were based on the annual report of the World Organization for Animal Health (OIE).^14^ Data are available for the year 2015 from the European Surveillance of Veterinary Antimicrobial Consumption (ESVAC), the US FDA, the Agricultural and Livestock Service, Chile (SAG), and the Ministry of Agriculture, Forestry and Fishery, Japan (MAFF).^14^ Of 62 countries with official data on antibiotic consumptions in 2015, 25 (40%) had data across all sectors (humans at community and hospital levels and animals). Those included Japan and 24 European countries.^14^^,^^15^ The figure is reproduced from www.antibioticfootprint.net under the terms of the Creative Commons Attribution 4.0. This figure appears in colour in the online version of *JAC* and in black and white in the print version of *JAC*.

**Figure 3.**
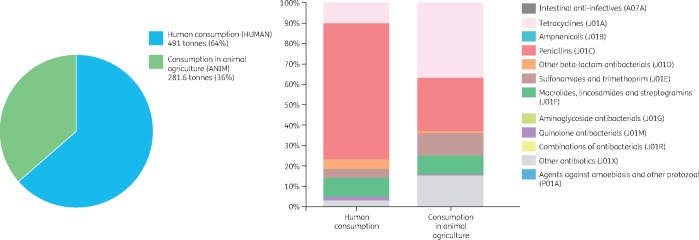
An example of the antibiotic footprint of a country based on antibiotic consumption in the UK in 2017. The country antibiotic footprint is defined here as the total amount of antibiotics consumed in the country. Total antibiotic consumption for human use and animal use was based on the UK One Health Report.^20^ The figure is reproduced from www.antibioticfootprint.net under the terms of the Creative Commons Attribution 4.0. This figure appears in colour in the online version of *JAC* and in black and white in the print version of *JAC.*

Similar to the carbon footprint, the antibiotic footprint of each country with official data could be presented and compared (Figure 2). This information would inform both policymakers and the community. For example, we might define a country’s antibiotic footprint as the total amount of antibiotics consumed in that country. The antibiotic footprint could be estimated by combining the total amount of antibiotics consumed by humans and animals in a given country (Figure 3). Consequently, the antibiotic footprint per capita (person) could also be estimated. While it is true that antibiotic use in animal agriculture would increase or decrease depending on the total amount of livestock being raised in the country, it is also true that countries that only import meat and do not raise any animals will not have antibiotics excreted from animals into the environment. This is why we propose using total consumption by country (Figure 3).

These data visualizations (Figures 2 and 3) can also prompt people to ask ‘How much antibiotic is being used in countries without official data?’. It is worth noting that official data on antibiotic consumption in many LMICs (including India and China) are currently not available.^15^ However, research evidence suggests that antibiotic consumption in human and animals in these countries is high.^18^^,^^19^

Clearly, crude comparisons of the antibiotic footprint do not take account of variations in populations, risk factors for infections and different farming practices. However, data can be used to prompt people to ask questions, such as ‘I didn’t know that so much antibiotic was used in food production—is this being reduced?’. For example, in 2013, total antibiotic consumption in the UK was 957 tonnes, of which 521 tonnes (55%) was for direct human consumption and 436 tonnes (45%) was for animal use.^20^ When compared with 2013, the total consumption for human use in 2017 decreased by 6% to about 491 tonnes, while the total antibiotic consumption for animal use in 2017 decreased by 35% to 282 tonnes (Figure 3).^20^ Of the 491 tonnes used in humans, it was estimated that 20% was used in the hospital sector and 80% in the community (primary care sector).^20^

Crude comparison of average consumption per capita could also prompt people to ask whether misuse and overuse of antibiotics occur. For example, worked out as an average per head of population in 2015, a person living in the UK is directly consuming twice as much antibiotic as a person living in the Netherlands (8.3 versus 3.3 g, respectively).^15^ This is supported by the comparison of DDD per 1000 inhabitants per day between the two countries (20.47 versus 9.78, respectively).^15^ It is unlikely that a higher incidence of bacterial infections in the human population accounts for this difference; differences in healthcare systems, patient expectations and attitudes to taking antibiotics are the likely major contributors. The large difference between average consumption of antibiotics per capita in the UK and Spain in 2015 (14.3 versus 72.6 g; Figure 2b) is largely because the total consumption of antibiotics in animals in the UK is much lower than that in Spain (395.1 versus 3027.7 tonnes, respectively). Data on mg/PCU suggest that livestock farming in Spain used about six times more antibiotics (per 1 kg of biomass) than livestock farming in the UK.^12^

National data for antibiotics discharged directly into the environment, for example by pharmaceutical companies, are still largely unavailable worldwide.^21^ However, research has shown that there can be considerable environmental contamination during antibiotic production in some countries.^21^ The AMR Industry Alliance (www.amrindustryalliance.org) is working to improve this. The quantity of environmental contamination could also be added to a country’s total consumption data where available.

An antibiotic footprint could also be calculated for individuals. As antibiotic use in humans varies by age, gender, local culture and individual attitude to taking antibiotics, etc., it would be informative to compare ourselves with other people in our own country, as well as other countries. Similar to on-line calculators for the carbon footprint, on-line individual calculators for antibiotic footprint could provide information, knowledge and recommendations about antibiotic consumption that could influence individual behaviour change, such as the impact of hand hygiene, vaccination and considered food choices.

The antibiotic footprint could be a tool to support global targets for reducing antibiotic consumption. The carbon footprint has been an important tool to support global targets to reduce carbon emissions. Progress in reducing global antibiotic consumption and that of individual countries is poorly described or defined. For example, colistin is currently considered as a last defence against some MDR infections. In 2016, the EU set a target for desirable levels of colistin use in food-producing animals between ≤5 and ≤1 mg/PCU.^22^ This goal is not yet agreed as a global target. In addition, official data for colistin consumption in LMICs are still largely unavailable. Setting targets for antibiotic consumption will be difficult because this combines the reduction of inappropriate use, whilst not discouraging appropriate use and access for those people and animals that need them. Nonetheless, it is possible that the antibiotic footprint concept could support implementation of future global targets.

## How do we reduce our antibiotic footprint?

People can reduce their own antibiotic footprint to a minimum by adjusting their activities and behaviour. This could include interventions to reduce infection risk such as ensuring uptake of available vaccinations, improving hand and food hygiene, and keeping healthy through following recommended exercise and dietary guidance.^23^ Diagnostic tests or scoring systems could also reduce inappropriate antibiotic prescribing (particularly for the common cold or acute sore throat using the low FeverPAIN score or Centor score).^24^ If the incidence of infectious diseases declines, opportunities for both appropriate and inappropriate antibiotic consumption in the whole population will also decline. Healthcare providers should continue to improve antibiotic stewardship and infection control in both hospitals and the community. Improved sanitation and water supplies in many developing countries will make a major difference.

Antibiotic use in food production can be lowered by promoting good animal husbandry standards. In 2017, UK supermarkets Asda, Waitrose and Marks & Spencer published farm-level antibiotic use data.^25^^,^^26^ This represents a major step in retailer transparency. Nonetheless, changing human behaviour is complex. Food labelling is contentious because food should not contain antibiotics at the point of sale, even if they have been used in the production process. Terms such as ‘antibiotic-free’ and ‘organic’ can be misleading, especially as there is no global agreement on their definition. Furthermore, animal welfare may be compromised where these terms are being used. Further studies and evaluations on communication and implementation of reducing overuse and misuse of antibiotics are needed. Licensing authorities should ensure that both the manufacture of active pharmaceutical ingredients and their assembly into antibiotic products do not cause environmental contamination.

The antibiotic footprint also has limitations. The availability of open-access data on national antibiotic consumption in human and animal sectors in LMICs is still limited. For the carbon footprint, different types of energy source may have different impacts on climate change, and a formula using emission factors (for example, for methane and ammonia) to standardize carbon emission is needed.^27–29^ Similarly, methods to compare antibiotic consumption in human and animal sectors and for different types of antibiotics will also be needed in the future. The effects of communication of the antibiotic footprint to people also need further testing.

## Conclusions

We propose that the antibiotic footprint could be one of a suite of tools to communicate information to the community on antibiotic usage. It could support global target setting to reduce the overuse and misuse of antibiotics in the future, both in people and in animals.
